# Precise Control over the Rheological Behavior of Associating Stimuli-Responsive Block Copolymer Gels

**DOI:** 10.3390/gels1020235

**Published:** 2015-12-07

**Authors:** Jérémy Brassinne, Flanco Zhuge, Charles-André Fustin, Jean-François Gohy

**Affiliations:** Institute of Condensed Matter and Nanosciences (IMCN), Bio- and Soft Matter (BSMA) Division, Université catholique de Louvain (UCL), Place L. Pasteur 1, Louvain-la-Neuve 1348, Belgium; E-Mails: jeremy.brassinne@uclouvain.be (J.B.); flanco.zhuge@uclouvain.be (F.Z.)

**Keywords:** supramolecular, stimuli-responsive, micellar gel, associating polymer, terpyridine, rheology

## Abstract

“Smart” materials have considerably evolved over the last few years for specific applications. They rely on intelligent macromolecules or (supra-)molecular motifs to adapt their structure and properties in response to external triggers. Here, a supramolecular stimuli-responsive polymer gel is constructed from heterotelechelic double hydrophilic block copolymers that incorporate thermo-responsive sequences. These macromolecular building units are synthesized via a three-step controlled radical copolymerization and then hierarchically assembled to yield coordination micellar hydrogels. The dynamic mechanical properties of this particular class of materials are studied in shear flow and finely tuned via temperature changes. Notably, rheological experiments show that structurally reinforcing the micellar network nodes leads to precise tuning of the viscoelastic response and yield behavior of the material. Hence, they constitute promising candidates for specific applications, such as mechano-sensors.

## 1. Introduction

“Smart” materials, *i.e.*, whose properties can be significantly changed in a controlled fashion by external triggers, are experiencing an unprecedented development over the last few years [1]. As a mirror of modern society, the practical demand for “smart” devices has overwhelmed all other forms of computing and communications in a very short time. They consist of systems that can operate to some extent interactively and autonomously to fulfill individual requirements. Analogously, “smart” materials have been designed to support a range of properties pertaining to use in various environments. In this regard, the most relevant focus having attracted much attention concerns “smart” polymer gels [2,3,4]. Due to their large, often macroscopic responses, they may indeed find numerous technological applications as mechano-sensors or soft actuators [5].

At the basis of “smart” materials lie intelligent polymer sequences or (supra-)molecular motifs having the ability to adapt their conformations and properties in response to external triggers, such as temperature [6,7,8,9], light [10,11,12] or pH [13,14,15]. Those variables can notably reverse the solvophilicity of synthetic macromolecules incorporating responsive groups, which pave the way for stimuli-sensitive materials. Another strategy to impart responsiveness to materials involves the use of secondary interactions, like hydrogen bonding [16,17], ionic interactions [18,19], π-stacking [20] or metal-ligand coordination [21]. They are indeed weaker, but dynamically more labile than primary covalent bonds [22,23], which impart adaptive properties to non-covalent assemblies [8,24].

In the field of environmentally-adaptive polymers, the thermo-sensitive poly(*N*-isopropylacrylamide) (PNIPAAm) constitutes an intensively-used polymer whose aqueous solutions show a lower critical solution temperature (LCST) around 32 °C [25]. Besides, many other thermo-responsive polymers have also demonstrated their applicability in the preparation of smart materials [6]. Among them, poly(2-(dimethylamino)ethyl methacrylate) (PDMAEMA) is particularly attractive, since it further shows pH responsiveness. By raising the hydrophilic nature of PDMAEMA through protonation of tertiary amino-groups [26], the overall hydrogen bonding ability of the macromolecules, as well as their electrostatic repellency are indeed increased, which leads to higher transition temperatures [26,27,28,29].

Although synthetically challenging, stimuli-responsive multi-block copolymers produce well-defined materials through the triggered or autonomous non-covalent associations of one or more blocks. In turn, they provide appealing flexibility for controlling the material micro-structure and physical properties through the application of proper stimuli [30,31,32]. In this context, our research has been focused on the synthesis of stimuli-responsive sequenced copolymers [33,34,35] and their self-assembly into “smart” materials with controlled structure and properties [36,37,38,39,40,41,42,43]. Our design strategy relies on a combination of metal-ligand and hydrophobic interactions, whose strength, density and dynamics can be adjusted depending on the material [36,37,38] and environmental [39,40,41] variables.

In the present paper, the purpose is to demonstrate the possibility of finely controlling the rheological behavior of this particular class of stimuli-responsive polymer gels. To this aim, a heterotelechelic double hydrophilic block copolymer is designed by means of controlled radical copolymerization techniques. This macromolecular building block is then hierarchically organized in aqueous media to yield a metallo-supramolecular micellar network. At its core, this work aims at studying in detail the rheological response of the self-assembled material in response to temperature changes.

## 2. Results and Discussion

In the last decade, the interest in polymers incorporating supramolecular motifs has dramatically risen, since they can easily find advanced applications as smart materials [44]. In the following, we describe the synthesis of a terpyridine end-capped polystyrene-*block*-poly(*N*-isopropylacrylamide)-*block*-poly(2-(dimethylamino)ethyl methacrylate) triblock terpolymer (PS-*b*-PNIPAAm-*b*-PDMAEMA-*b*-tpy) and its hierarchical assembly into a coordination micellar network. Rotational rheometry is then used as a characterization tool to probe the thermo-mechanical properties of the supramolecular hydrogel.

### 2.1. Synthesis of Functional Building Block

Among coordination motifs, the 2,2';6',2''-terpyridine ligand is particularly attractive, since it forms stable *bis*-complexes in combination with various transition metal ions [45,46]. Being the most prominent representative of its family [47,48], this *N*-heteroaromatic ligand can be easily introduced into macromolecular architectures by post-modification [49,50,51], or via the use of modified comonomers [52,53,54,55] or chain initiators [56,57]. More recently, the use of terpyridine-modified chain transfer agents (CTA) has been developed as a straightforward approach toward functional block copolymers [33,34]. In this study, a dithiobenzoate is selected due to its compatibility with various functional monomers [58], affording the possibility to sequentially copolymerize methacrylate, acrylamide and styrene monomers [59,60]. This control agent is derived from commercially available 4-(4-cyanopentanoic acid) dithiobenzoate (CPAD) according to a procedure reported elsewhere [34].

**Figure 1:**
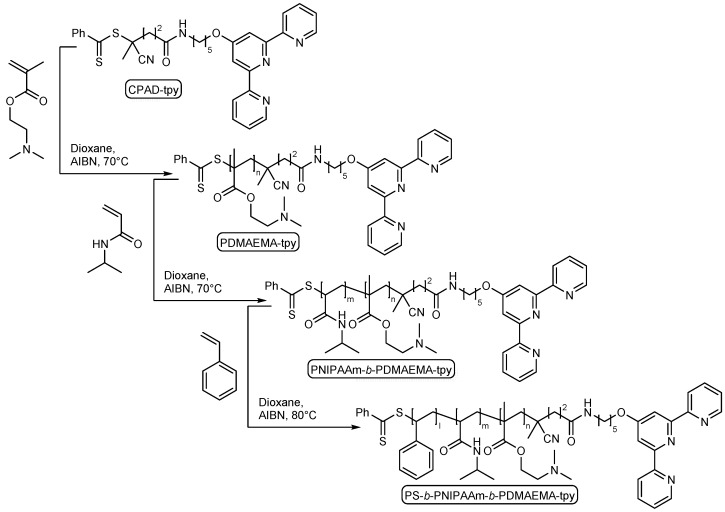
Sequential reversible addition-fragmentation chain transfer (RAFT) copolymerization leading to polystyrene-*block*-poly(*N*-isopropylacrylamide)-*block*-poly(2-(dimethylamino)ethyl methacrylate) triblock terpolymer (PS-*b*-PNIPAAm-*b*-PDMAEMA-*b*-tpy). CPAD, 4-(4-cyanopentanoic acid) dithiobenzoate; AIBN, 2,2'-azobis(isobutyronitrile).

The synthesis of the PS-*b*-PNIPAAm-*b*-PDMAEMA-tpy triblock copolymer is sequentially achieved by reversible addition-fragmentation chain transfer (RAFT) polymerization, as depicted in Figure 1. This copolymer is designed to produce self-assembled hydrogels that would combine the association strength of polystyrene stickers and metal-terpyridine complexes, with the stimuli-responsiveness of both PNIPAAm and PDMAEMA blocks. Following a procedure reported elsewhere [34], a terpyridine end-capped double hydrophilic PNIPAAm_73_-*b*-PDMAEMA_103_ copolymer is first synthesized, the number in subscript referring to the average degree of polymerization of each block. In a third step, styrene is polymerized in the presence of the PNIPAAm-*b*-PDMAEMA-tpy copolymer as macro-CTA and 2,2'-azobis(isobutyronitrile) (AIBN) as a source of primary radicals (Figure 1). In order to ensure control over the polymerization process, the initial ratio between styrene, macro-CTA and AIBN is set to 3000:7.5:1. In practice, the reaction is conducted at 80 °C in dry 1,4-dioxane and stopped at a predetermined time interval to afford a polystyrene block of a few units. In our strategy, the length of the PS segment is deliberately kept short to allow direct dissolution of the triblock copolymer in aqueous media.

**Figure 2:**
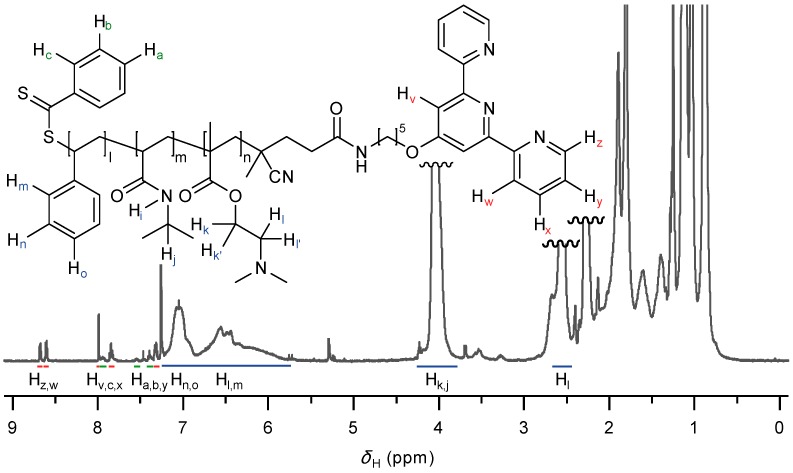
^1^H-NMR spectrum of pure PS-*b*-PNIPAAm-*b*-PDMAEMA-tpy triblock terpolymer in deuterated chloroform as the solvent.

After the polymerization step, the synthesized triblock copolymer is purified by precipitation of the crude reaction mixture, followed by isolation of the precipitate and subsequent drying. The composition of the copolymer is determined to be PS_12_-*b*-PNIPAAm_73_-*b*-PDMAEMA_103_-tpy by means of proton nuclear magnetic resonance (^1^H-NMR) spectroscopy. As shown in Figure 2, the ^1^H-NMR spectrum analysis reveals characteristic broad signals of polystyrene and further attests to the presence of terpyridine ligand in the chain architecture. Practically, the length of the polystyrene block is estimated from the ratio between the peak area of PS aromatic protons and isopropyl/aliphatic ester protons of the PNIPAAm and PDMAEMA block around 4 ppm.

**Figure 3:**
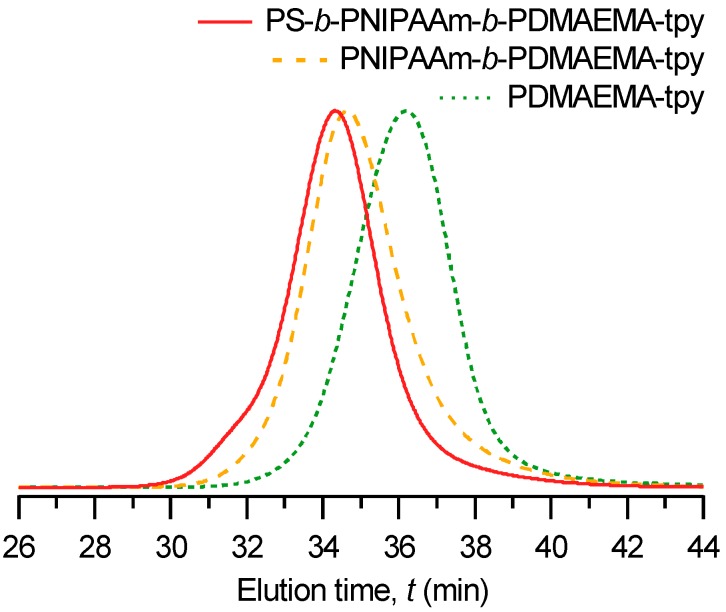
Size exclusion chromatography (SEC) elugrams of PDMAEMA-tpy homopolymer, derived PNIPAAm-*b*-PDMAEMA-tpy diblock copolymer and PS-*b*-PNIPAAm-*b*-PDMAEMA-tpy triblock terpolymer.

As shown in Figure 3, the chain extension of PNIPAAm-*b*-PDMAEMA-tpy macro-CTA into PS-*b*-PNIPAAm-*b*-PDMAEMA-tpy triblock terpolymers is further evidenced by size exclusion chromatography (SEC). This analysis indeed shows a significant shift of the SEC trace to lower elution times after polymerization of the third block, indicating an increase in molar masses. Last, but not least, the SEC analysis reveals still narrow molar mass dispersity (*Ð* = 1.29), attesting to the control over the polymerization process.

### 2.2. Self-Assembly into Metallo-Supramolecular Hydrogel

**Figure 4:**
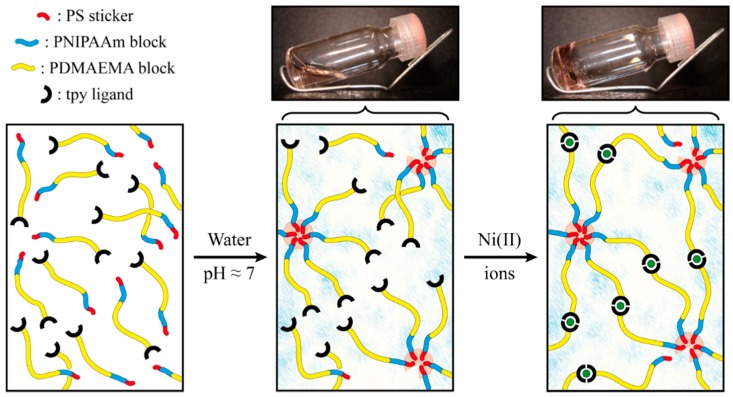
Schematic representations and illustrations of the hierarchical assembly of PS-*b*-PNIPAAm-*b*-PDMAEMA-tpy associating copolymers into a coordination micellar gel.

The synthesized heterotelechelic associating copolymer is then used as precursor of a metallo-supramolecular hydrogel. As schematized in Figure 4, the first level of assembly is achieved upon direct dissolution of the triblock copolymer in ultra-pure water, which presumably leads to the formation of micelles due to the high incompatibility of polystyrene with aqueous media. Thanks to the shortness of this hydrophobic block, the copolymer is found to be easily dispersible in water at low to room temperature and a concentration ranging in the semi-dilute regime. The pH of the solution is adjusted around neutral to ensure a good solubility of the PDMAEMA block, which further avoids its co-precipitation above the LCST of the PNIPAAm block [34]. At this point, a clear free-flowing concentrated solution is obtained, and no gelation occurred, which can be explained by the lack of entanglement between coronal chains of neighboring micelles.

**Figure 5:**
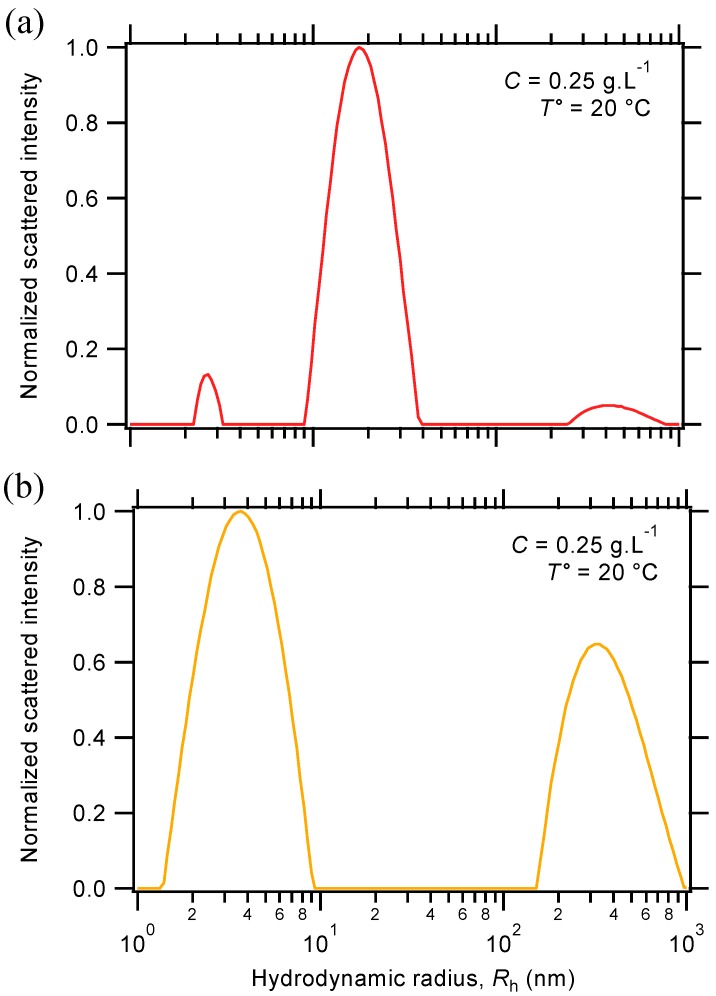
Distribution of hydrodynamic radii of (**a**) the PS-*b*-PNIPAAm-*b*-PDMAEMA-tpy triblock terpolymer and (**b**) the parent PNIPAAm-*b*-PDMAEMA-tpy diblock copolymer in aqueous solution.

In practice, the formation of triblock copolymer micelles is tested by dynamic light scattering (DLS) measurements, which provide information about the size distributions of samples by the analysis of scattered light intensity. As shown in Figure 5a, the distribution of hydrodynamic radii (*R*_h_) essentially reveals the presence of micellar nanostructures in the triblock terpolymer solution, with an apparent radius size around 18 nm. Small proportions of isolated chains (unimers), as well as aggregates are also evidenced in the investigated concentration range, respectively around a few and a few hundred nanometers in size. To provide further insight into the micellization process, the solution behavior of the PS-*b*-PNIPAAm-*b*-PDMAEMA-tpy associating terpolymer is compared to the one of the parent diblock copolymer. As shown in Figure 5b, DLS measurement on the PNIPAAm-*b*-PDMAEMA-tpy diblock copolymer solution mainly reveals the presence of an isolated chain, accompanied by larger aggregates that could in fact arise from slow diffusion modes of the polyelectrolyte. However, the micellar nano-objects are no longer detected, which also explains the very low intensity of light scattered by the diblock copolymer solution. On the whole, these experiments indicate that the formation of micelles is indeed driven by the aggregation of the PS segment in aqueous triblock terpolymer solution. Furthermore, the weak dependence of the DLS signal as a function of the scattered angle strongly suggests the formation of spherical micelles, as expected, given the balance between the hydrophobic and hydrophilic blocks.

As a second level of assembly, the stoichiometric amount of half an equivalent of transition metal ions dissolved in water is added to the concentrated micellar solutions to reach a final weight-to-volume fraction (*C*) of 5% *w*/*v*. In this concentration regime, the presence of metal ions in the media would result in inter-micellar complexation between the segregated PS nano-domains formed in the aqueous environment (Figure 4). Of course, the choice of the metal ion used for the terpyridine complexation constitutes a powerful means to control the mechanical properties of terpyridine-based gels [61,62]. This consideration was addressed in our previous study, where the effect of different transition metal ions was tested on the rheological properties of metallo-supramolecular micellar hydrogels [36]. In this study, nickel(II) ions are selected, since they afford one of the more stable *bis*-complexes in combination with the terpyridine ligand [63], thereby providing efficient bridges towards the formation of a supramolecular network. In practice, the establishment of a percolated network structure is evidenced by the tube inversion test at room temperature. As shown in Figure 4, the initially free-flowing concentrated solution of PS-*b*-PNIPAAm-*b*-PDMAEMA-tpy copolymers indeed turns into a free-standing supramolecular gel upon the addition of metal ions, which occurs within minutes.

**Figure 6:**
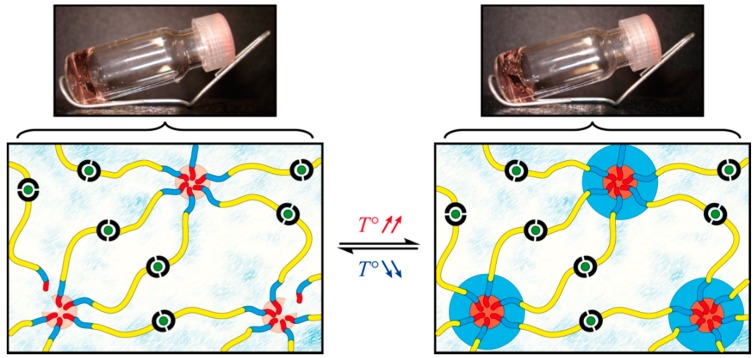
Schematic representations and illustrations of the thermo-response of the PS-*b*-PNIPAAm-*b*-PDMAEMA-tpy triblock copolymer gel.

Structurally speaking, a free-standing supramolecular hydrogel is obtained thanks to the aggregation of hydrophobic polystyrene segments that provide branching points within the supramolecular network [36,40]. Upon heating, the structure of the triblock micellar network is expected to be further reinforced by the collapse of PNIPAAm segments onto micellar nodes, as illustrated in Figure 6. However, no phase separation is visually observed when heating the PS-*b*-PNIPAAm-*b*-PDMAEMA-tpy hydrogel, even well above the LCST of the PNIPAAm block. Actually, the partial collapse of coronal chains would result in the formation of three-layer micelles with a hydrophobic PS core, a collapsed PNIPAAm shell and a remaining PDMAEMA corona (Figure 6). In this picture, the soluble PDMAEMA segment in the network architecture ensures the swelling of the gel phase, in a temperature range that exceeds the upper solubility limit of the PNIPAAm block. Given the characteristics of the triblock copolymer, a core–shell-corona structure is realistically expected for the micelles at elevated temperature. Nevertheless, the actual morphology that the microphase-separated structures may adopt in the gel phase remains an open question that would require deeper analyses.

### 2.3. Characterization of the Rheological Response

As the main characterization tool, rotational shear rheometry is conducted on the PS-*b*-PNIPAAm-*b*-PDMAEMA-tpy hydrogels to elucidate the hypothetical effect of temperature (*T°*) on their rheological behavior. This technique allows determining the fraction of shear energy that is stored in elastic distortions of the polymer network, as measured by the storage modulus (*G'*). Complementary to this, the loss modulus (*G''*) measures the fraction of deformation energy that is dissipated due to relaxations that occur on the timescale of the experiment. Being intimately interrelated, the magnitude of those moduli is thus affected by each relaxation process of the polymer network, whose rates are determined by the molecular mechanism involved. Information about the structure and dynamics of the material can be thus accessed by following the evolution of dynamic moduli while varying the frequency and amplitude of the imposed stress.

In parallel, additional information about the structures of the assembly can be obtained in steady shear flow measurements. This complementary approach allows monitoring the equilibrium flow by maintaining a constant stress (*σ*) or shear rate ($\dot{\gamma }$) for a sufficient time in one direction to allow dynamic equilibrium to be achieved in the fluid. In particular, monitoring the material viscosity (*η*) as a function of the shear rate allows evaluating the resistance of the material to being deformed, which is crucial for processing.

In the following, the evolution of dynamic storage and loss moduli upon thermal variations is first followed in the form of temperature sweeps, at a given oscillatory frequency (*ω*) and low stress (*σ*_0_) or strain (*γ*_0_) amplitude. Then, frequency sweeps are performed under small amplitude oscillatory shear, over a temperature range that covers the phase transition of the PNIPAAm block. After that, the non-linear viscoelastic response of the gel is investigated by amplitude strain sweeps operating at different temperatures. Finally, steady shear flow measurements are conducted on the supramolecular gel, below and above the phase transition temperature.

#### 2.3.1. Oscillatory Temperature Sweep

**Figure 7:**
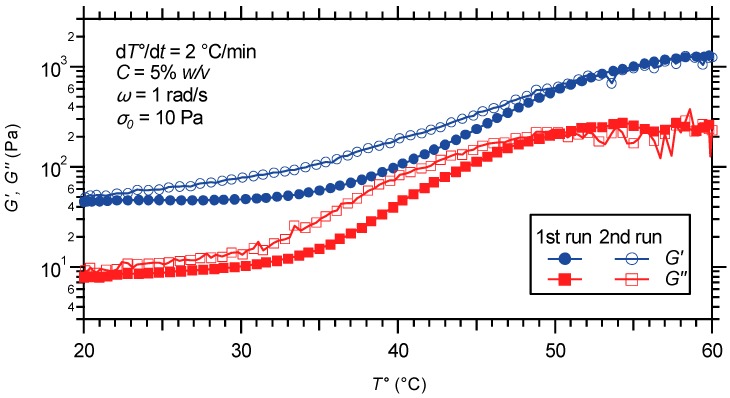
Temperature dependence of dynamic moduli for a hydrogel prepared from the PS-*b*-PNIPAAm-*b*-PDMAEMA-tpy copolymer and Ni(II) ions: first run upon heating; second run upon cooling.

Due to an enhanced relaxation of the transient cross-links, supramolecular gels often suffer a loss in their mechanical properties when tested at elevated temperatures [8,64]. In sharp contrast, the PS-*b*-PNIPAAm-*b*-PDMAEMA-tpy hydrogel shows a strengthened viscoelastic response to shear as the temperature increases, as attested by dynamic temperature sweeps (Figure 7). In these experiments, the evolution of dynamic storage and loss modulus against temperature is monitored as an indication of the gel strengthening. During measurements, the temperature is varied between 20 and 60 °C (first run), followed by cooling at the same rate immediately after heating (second run). Each temperature ramp is performed at a low stress amplitude of 10 Pa, a fixed frequency of 1 rad/s, with a heating/cooling rate of 2 °C/min.

Under ambient conditions, the gel strength results from the aggregation of PS segments into hydrophobic domains that are further bridged by metal-ligand associations, thereby allowing elastic stretching. Above a certain temperature, core-shell-corona micelles are formed with the PS blocks forming micellar cores and the PNIPAAm segments constituting the shell layer. This temperature-induced transition, centered at 40 °C, stems from the solution behavior of the thermo-sensitive middle block of the triblock copolymer. When the temperature is raised above the LCST of PNIPAAm segments, they undergo a hydration-to-dehydration transition that reinforces the network structure, as illustrated in Figure 6. For both *G'* and *G''*, the thermal transition forms a continuum extending over almost the entire investigated temperature range, with a net increase in moduli approaching one and a half orders of magnitude. Then, decreasing temperature dissolves the PNIPAAm blocks and weakens the network, resulting in the reverse transition with almost no hysteresis (Figure 7).

#### 2.3.2. Oscillatory Frequency Sweep

**Figure 8:**
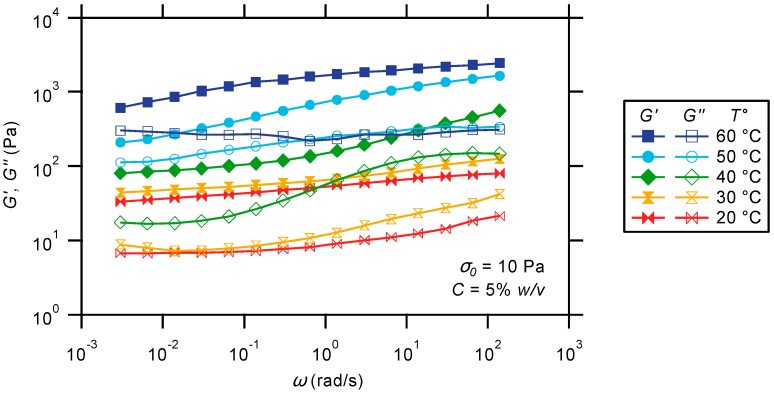
Frequency dependence of dynamic moduli for a hydrogel prepared from the PS-*b*-PNIPAAm-*b*-PDMAEMA-tpy copolymer and Ni(II) ions, at different temperatures.

To get further insight into the thermo-responsive behavior of PS-*b*-PNIPAAm-*b*-PDMAEMA-tpy triblock copolymer gels, frequency sweeps are conducted using a low stress amplitude of 10 Pa, at selected temperatures covering the strengthening transition range (Figure 8). Under ambient conditions, a rubber-like behavior is observed for the triblock copolymer gel, as indicated by the weak frequency-dependent plateaus in both moduli over the entire investigated frequency range. This signature evidences that neither the micellar cores nor the metal-ligand bridges between them relax on the experiment timescale. The absence of core relaxation can be attributed to the glassy nature of the polystyrene association. Indeed, block copolymer micelles having glassy cores are known to be kinetically frozen and are thus expected to have very slow unimer exchange kinetics [65]. As reported by several authors [66,67], the high energy barrier for unimer exchange between micelles having cores made of even short polystyrene segments leads to negligible exchange of chains at ambient temperature. In addition, the large incompatibility between hydrophobic segments and polyelectrolyte corona creates high interfacial tensions that are sufficient to freeze micellar aggregates, even above the glass transition temperature [68]. In parallel, the apparent inertness of coordination bridges is in good agreement with the exchange rates reported for metal–terpyridine bonds [45,46,69]. In this regard, Ni(II) ions are expected to form long-lifetime complexes in combination with the terpyridine ligand. This particular stability has been used in the strategic formation of metallo-supramolecular polymers with high structural integrity [34,63,70,71] or metallo-supramolecular networks with a delayed terminal relaxation [36,40].

In agreement with the temperature sweep, the plateaus in moduli further increase by around one and a half orders of magnitude when the temperature is increased to 60 °C. During the transition, the increase in moduli is however more pronounced in the high-frequency regime than in the low-frequency region, especially around 40 °C. Although a maximum in *G''* is not observed, the presence of an apparent fast relaxation process is postulated to account for the decrease in *G'* in this temperature range. Experimentally, the presence of multiple relaxation modes in thermo-rheologically-complex materials indeed leads to frequency dependence that does not always show a maximum in *G''*, especially when the relaxation processes are not well-defined or resolved. At higher temperatures, both *G'* and *G''* finally tend to show again much weaker frequency dependencies. Although unconventional, attempts are made to construct a master curve using a negative temperature shift factor, but result in a poor superposition of the curves compared to what is expected for thermo-rheologically-simple materials. Intuitively, the construction of a master curve is based on the assumption that the material behavior is thermo-rheologically simple; otherwise, the time-temperature superposition principle is not applicable. Here, this assumption is not verified, since the viscoelastic material functions are dramatically altered upon heating. Indeed, an increasing temperature not only affects the relaxation spectra of the material, but mainly causes the precipitation of the PNIPAAm blocks.

To explain the thermal transition in moduli, it is assumed that the relaxation being observed at intermediate temperatures corresponds to local and diffusional motions of PNIPAAm segments. At a low temperature, relaxation of the chain segments in solution is extremely prompt due to micro-Brownian motions or detachment from the weakly-associated micellar nodes, which do not contribute to the modulus. Being part of the fast relaxation spectrum of the material, these motions are not directly accessible by experiment, since they involve extremely short time scales and low activation energy barriers. Upon heating, the collapse of PNIPAAm blocks onto preformed micellar cores progressively restricts the motions of chain segments in solution. In turn, the decreasing degree of motional freedom and reinforcement of hydrophobic nodes, caused by intra- and inter-chain association of PNIPAAm in the collapsed state, results in a gradual shift of this relaxation mode to lower frequencies. At elevated temperatures, the advanced dehydration of the PNIPAAm block leads to a dramatic increase in the conformational rigidity of the network, therefore disabling the motions of PNIPAAm segments, as schematized in Figure 6.

Of course, the collapse of the PNIPAAm blocks also inevitably results in a non-trivial change in the structure of the material: at low temperature, the hydrogel presents the characteristic structural features of telechelic associating networks, each building elements bearing discrete associating units at each chain end; at an elevated temperature, the structure of the material evolves to the one of micellar gels, consisting of segregated nano-objects that are inter-connected by metal-ligand bridges. This transition can be plausibly accompanied by a modification in the rheology of the material structural elements or a variation in the number of elastically-active chains between them, occurring at a specific or over multiple assembly scales. In this regard, it is known that the rheological behavior of micelles is significantly influenced by their shape [41,72,73,74], which is in turn dictated by the hydrophobic-hydrophilic balance in the block copolymers and the nature of the solvating media. However, this situation is fundamentally encompassed by considering the overall change in the dynamic and number of network active chains, or network elements, in the multiscale assembled material.

#### 2.2.3. Oscillatory Strain Sweep

**Figure 9:**
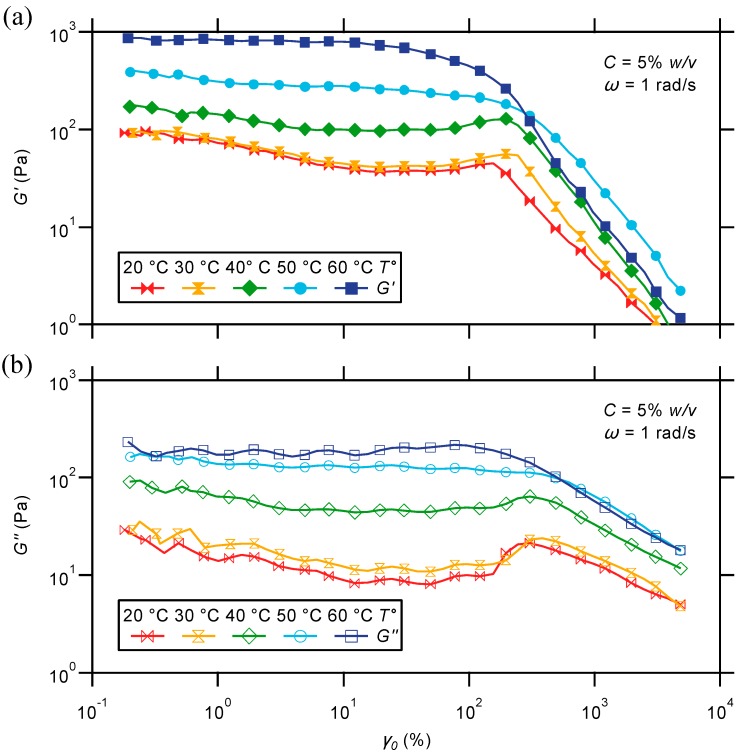
Strain dependence of (**a**) storage and (**b**) loss moduli for a hydrogel prepared from the PS-*b*-PNIPAAm-*b*-PDMAEMA-tpy copolymer and Ni(II) ions, at different temperatures.

To further look into the gel characteristics, amplitude strain sweeps are conducted on the triblock copolymer gel at various temperatures and a given frequency of 1 rad/s. As illustrated in Figure 9, the studied material displays a long linear viscoelastic response over the whole covered temperature range. Under small amplitude oscillatory shear, moduli indeed remain nearly invariant with respect to strain amplitude, indicating no breakup of the network structure. At larger deformations, both moduli drop dramatically with strain on the sample, which is highly characteristic of a non-linear response. Interestingly, the yield of the materials is observed around comparable strain amplitudes in nearly the entire temperature range. In practice, a significant deviation to lower yield strain is only observed at a temperature of 60 °C.

The dynamic strain sweep study also reveals significant thermal variations in the behavior of the gels at the limit of the linear range (Figure 9). At low temperatures, a hardening response to strain is observed prior to yielding that is particularly marked in the loss modulus. As suggested by Tam and coworkers [75], such a behavior may result from the incorporation of finite-sized aggregates into the percolated hydrogel, which is possible due to reorganization of the weakly-associated network structure. As the temperature is increased, the collapse of PNIPAAm segments onto micellar nodes is thought to reduce the tolerance and flexibility of the same structure against high shear deformation. As a consequence, strain softening is only observed above 40 °C, as shown in Figure 9.

#### 2.2.4. Steady Shear Viscometry

**Figure 10:**
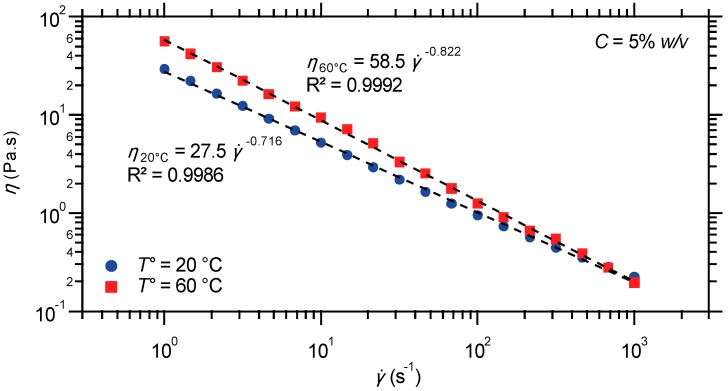
Shear rate dependence of steady shear viscosity for a hydrogel prepared from the PS-*b*-PNIPAAm-*b*-PDMAEMA-tpy copolymer and Ni(II) ions, at different temperatures.

Last, but not least, steady shear flow experiments are performed on the triblock terpolymer gel, below and above the phase transition temperature measured in oscillatory shear. In practice, the apparent viscosity of the material is monitored as a function of the rate at which it is sheared, thereby providing important information about processing. Indeed, the viscosity is the measure of a fluid’s internal flow resistance, which may sensibly vary when the material is sheared. As reported in Figure 10, the steady shear analysis has taken the data between 1 and 1000 s^−1^ shear rates, at 20 and 60 °C. Fundamentally, the results reveal that the supramolecular polymer gel is highly shear thinning, *i.e.*, its viscosity decreases with shear rate, at both temperatures. At a 1-s^−1^ shear rate, the material has an apparent viscosity order of magnitude higher than water. As the shear rate is increased, the apparent viscosity however decreases dramatically, which indicates the excellent processability of the gels. Since the data show linearity in the double-logarithmic plot, the viscosity dependence can be described by a power law: *η* = K. ${\dot{\gamma }}^{n-1}$, with shear-thinning indexes, *n*, close to zero.

At 60 °C, the material only shows a slight increase of apparent viscosity in the whole investigated shear rate range, which might be in fact due to non-negligible evaporation at this temperature. However, the shear-thinning behavior is more pronounced than under ambient conditions, as indicated by the slope in the viscosity-shear rate graph (Figure 10). Fundamentally, this behavior can be rationalized by the decreasing volume fraction of polymer chains in solution at elevated temperature due to the collapse of the PNIPAAm segments [76]. In addition, this observation strongly supports the formation of well-dispersed spherical core-shell network nodes upon heating, as hypothetically depicted in Figure 6. Indeed, a transition in micelle morphology to, e.g., rods or continuous phases, would have resulted in a large increase in viscosity and a hindered flow due to additional contact between the extended hydrophobic domains [77], which is not observed here.

## 3. Conclusions

In conclusion, we described here the synthesis and self-assembly of a heterotelechelic double hydrophilic block copolymer, one extremity being ended by a short associating sticker, the other bearing a chelating ligand. Precisely, a terpyridine end-functionalized polystyrene-*block*-poly(*N*-isopropylacrylamide)-*block*-poly(2-(dimethylamino)ethyl methacrylate) triblock terpolymer was synthesized via sequential controlled radical copolymerization, using a modified chain transfer agent. Through the hydrophobic and coordination terminal moieties, the associating copolymer was hierarchically assembled into a supramolecular network with a precisely-controlled architecture. The gelation was induced by the formation of metal-ligand bridges between micellar nodes resulting from the aggregation of polystyrene segments in aqueous media. Due to the tailored network structure, these transient nodes can be shielded by the thermo-induced collapse of the PNIPAAm blocks, which opened a way to finely tune the mechanical properties of the gel.

The shear viscosity, frequency- and strain-dependences of the viscoelastic properties of the gel were investigated as a function of the temperature. Notably, results suggested that the high degree of freedom of the PNIPAAm segment in the hydrated state allowed partial relaxation of mechanical stress. This wasted contribution to the elastic response was however modulated by the solubility of the middle block at higher temperatures. By ultimately suppressing the segmental relaxation, the collapse of the PNIPAAm block also led to stronger hydrophobic associations that were less easily disrupted by mechanical forces. Finally, the response of the gels under large oscillatory shear was sensibly varied across the investigated temperature range. Precisely, an overshoot in both moduli was observed below the collapse transition temperature of the PNIPAAm segment, while the same materials exhibited strain thinning only at higher temperatures.

Combining valuable mechanical properties and the ability to modulate them via temperature changes, the investigated material constitutes a promising candidate for specific applications, such as mechano-sensors or actuators. In this continuity, the influence of several parameters, like, e.g., the length of the different blocks and the pH of the media, will be investigated on the dynamic mechanical properties of this particular class of stimuli-responsive gels. In parallel, future works will focus on the structural characterization of the triblock terpolymer hydrogel. In this regard, the small-angle neutron scattering technique can be identified as a powerful characterization technique for monitoring the change in material structure along the thermal transition.

## 4. Experimental Section 

### 4.1. Materials

All chemicals were purchased from Acros (Acros Organics Belgium, Geel, Belgium) or Aldrich (Sigma-Aldrich, Belgium, Diegem, Belgium) and were of the highest purity grade. All chemicals were used as received unless otherwise specified. 2,2'-Azobis(isobutyronitrile) was recrystallized from methanol. Styrene and 2-(dimethylamino)ethyl methacrylate were dried and vacuum-distilled over calcium hydride. *N*-isopropylacrylamide was recrystallized from *n*-hexane and dried overnight in a vacuum oven at 35 °C prior to use. Dichloromethane and 1,4-dioxane were distilled over calcium hydride. NiCl_2_ transition metal salt was dried before use.

### 4.2. Instrumentation

All ^1^H nuclear magnetic resonance spectra were recorded on a Bruker 300 MHz Avance II spectrometer (Bruker Belgium, Brussels, Belgium) in deuterated solvents containing tetramethylsilane as an internal standard. Chemical shifts (*δ*) were reported in parts per million downfield from the internal standard. Size exclusion chromatography was performed in *N*,*N*-dimethylformamide containing 2.5 mM NH_4_PF_6_ to determine molecular weight distributions with respect to polystyrene standards (Polymer Standard Service (PSS), Mainz, Germany). The measurements were carried out on a system composed of two PSS Gram columns (100 and 1000 Å) connected to a Waters 410 differential refractive index detector operating at 0.5-mL/min flow rate and a temperature of 35 °C. Dynamic light scattering experiments were performed on a Malvern CGS-3 apparatus equipped with a He–Ne laser with a wavelength of 632.8 nm and a thermostat. The size distribution histograms of the self-assembled nanostructures were obtained using the constrained regularization method for inverting data (CONTIN), which is based on an inverse-Laplace transformation of the data. Shear rheological experiments were performed on a Kinexus Ultra (Malvern Instruments, Hoeilaart, Belgium) rheometer equipped with a heat exchanger and modified with a solvent trap. Measurements were carried out using a 20-mm plate-plate geometry, in a water-saturated atmosphere, in order to minimize evaporation of the solvent. The gap was adjusted between, so that the geometry was completely filled. Normal forces were checked to be relaxed prior to any measurement.

### 4.3. Synthesis of PDMAEMA_103_-tpy

CPAD-tpy (50.3 mg, 84 μmol), DMAEMA (4.27 mL, 25.3 mmol) and AIBN (2.8 mg, 17 μmol) were dissolved in 1,4-dioxane (9.1 mL). The solution was degassed three times by freeze-pump-thaw, filled with argon and stirred in a preheated paraffin oil bath at 70 °C. After 6 h, the polymerization was stopped by placing the Schlenk tube into liquid nitrogen. The monomer conversion was evaluated around 40% from ^1^H-NMR integration. The homopolymer solution was precipitated twice into a 20-times excess of cold *n*-hexane. The precipitate was isolated by centrifugation at 2000 rpm and dried under vacuum at room temperature to afford a red semi-solid.

^1^H-NMR (300 MHz, CDCl_3_) *δ*_H_: 8.69 (d, 2H), 8.61 (d, 2H), 8.01 (s, 2H), 7.91 (t, 2H), 7.85 (td, 2H), 7.56 (t, 1H), 7.38 (t, 2H), 7.33 (dd, 2H), 5.70 (br, 1H), 4.24 (t, 2H), 4.07 (br, 206H), 3.31 (q, 2H), 2.54 (t, 2H), 2.53 (br, 206H), 2.52 (m, 2H), 2.26 (br, 618H), 1.88 (q, 2H), 1.88–1.80 (br, 206H), 1.03–0.87 (br, 309H), 1.78 (q, 2H), 1.70 (q, 2H), 0.88 (s, 3H).

*M*_n_ (SEC) = 20,000 g·mol^−1^, *M*_w_ (SEC) = 23,950 g·mol^−1^, *M*_w_/*M*_n_ (SEC) = 1.20; *M*_n_ (NMR) = 16,750 g·mol^−1^.

### 4.4. Synthesis of PNIPAAm_73_-b-PDMAEMA_103_-tpy

PDMAEMA-tpy (750 mg, 45 μmol), NIPAAm (1.81 g, 16 mmol) and AIBN (1.3 mg, 8 μmol) were dissolved in 1,4-dioxane (7.44 mL). The solution was degassed three times by freeze-pump-thaw, filled with argon and stirred in a preheated paraffin oil bath at 70 °C. After 2 h, the polymerization was stopped by placing the Schlenk tube into liquid nitrogen. The monomer conversion was evaluated around 20% from ^1^H-NMR integration. The copolymer solution was precipitated five times into a 10-times excess of *n*-hexane. The precipitate was isolated by centrifugation at 2000 rpm and dried under vacuum at room temperature to afford a pale red solid.

^1^H-NMR (300 MHz, CDCl_3_) *δ*_H_: 8.69 (d, 2H), 8.61 (d, 2H), 8.01 (s, 2H), 7.91 (d, 2H), 7.85 (td, 2H), 7.56 (tt, 1H), 7.38 (t, 2H), 7.33 (dd, 2H), 6.30 (br, 73H), 5.70 (br, 1H), 4.24 (t, 2H), 4.07 (br, 206H), 3.99 (br, 73H), 3.31 (q, 2H), 2.54 (t, 2H), 2.53 (br, 206H), 2.52 (m, 2H), 2.26 (br, 618H), 2.08 (br, 73H), 1.88 (q, 2H), 1.88–1.80 (br, 206H), 1.82–1.62–1.33 (br, 146H), 1.12 (br, 438H), 1.03-0.87 (br, 309H), 1.78 (q, 2H), 1.70 (q, 2H), 0.88 (s, 3H).

*M*_n_ (SEC) = 29,650 g·mol^−1^, *M*_w_ (SEC) = 36,450 g·mol^−1^, *M*_w_/*M*_n_ (SEC) = 1.23; *M*_n_ (NMR) = 25,000 g·mol^−1^.

### 4.5. Synthesis of PS_12_-*b*-PNIPAAm_73_-*b*-PDMAEMA_103_-tpy

PNIPAAm-*b*-PDMAEMA-tpy (200.5 mg, 8 μmol), styrene (368 μL, 3.2 mmol) and AIBN (0.175 mg, 1.05 μmol) were dissolved in 1,4-dioxane (1.549 mL). The solution was degassed three times by freeze-pump-thaw, filled with argon and stirred in a preheated paraffin oil bath at 80 °C. After 1 h, the polymerization was stopped by placing the Schlenk tube into liquid nitrogen. The copolymer solution was precipitated three times into a 10-times excess of *n*-hexane. The precipitate was isolated by centrifugation at 2000 rpm and dried under vacuum at room temperature to afford a pale red solid.

^1^H-NMR (300 MHz, CDCl_3_) *δ*_H_: 8.69 (d, 2H), 8.61 (d, 2H), 8.01 (s, 2H), 7.91 (d, 2H), 7.85 (td, 2H), 7.56 (tt, 1H), 7.38 (t, 2H), 7.33 (dd, 2H), 7.05 (br, 24H), 6.95 (br, 12H), 6.50 (br, 24H), 6.30 (br, 73H), 5.70 (br, 1H), 4.24 (t, 2H), 4.07 (br, 206H), 3.99 (br, 73H), 3.31 (q, 2H), 2.54 (t, 2H), 2.53 (br, 206H), 2.52 (m, 2H), 2.30–1.70 (br, 12H), 2.26 (br, 618H), 2.08 (br, 73H), 1.88 (q, 2H), 1.88–1.80 (br, 206H), 1.82–1.62–1.33 (br, 146H), 1.12 (br, 438H), 1.03-0.87 (br, 309H), 1.78 (q, 2H), 1.70 (q, 2H), 1.90–1.30 (br, 24H), 0.88 (s, 3H).

*M*_n_ (SEC) = 31,300 g·mol^−1^, *M*_w_ (SEC) = 40,400 g·mol^−1^, *M*_w_/*M*_n_ (SEC) = 1.29; *M*_n_ (NMR) = 26,250 g·mol^−1^.

### 4.6. Sample Preparation

The hydrogels were prepared by dissolving given amounts of block copolymer in ultrapure water. The sealed reaction vessels was placed in a fridge and shaken periodically to form a homogeneous concentrated solution after a few days. The pH of the solution was adjusted via the addition of hydrochloric acid aqueous solution of a given molarity. The gel was then readily obtained by adding the stoichiometric amount of half an equivalent of transition metal ions (with respect to the terpyridine content) dissolved in a defined amount of ultra-pure water to the concentrated solution. Lastly, the reaction vessel was placed again in the fridge over three days to ensure homogenization and stabilization of the material. The final concentration of copolymer in the sample was 5% *w/v*.

### 4.7. Loading and Testing Protocol

For each test, around 50 μL of the material were loaded onto the stationary bottom plate of the rheometer preheated at 20 °C. By stepwise lowering the gap between the two plates, the samples were compressed and forced to spread over the geometry, so that the gap was completely filled. Equilibration of the samples was followed by monitoring the evolution of normal force, storage and loss moduli with time, under small amplitude oscillatory shear. Rheological tests were started when both moduli reached constant values and the normal force had relaxed to <0.05 N.

## References

[1] Dai L. (2003). Intelligent Macromolecules for Smart Devices.

[2] Ahn S.K., Kasi R.M., Kim S.C., Sharma N., Zhou Y.X. (2008). Stimuli-responsive polymer gels. Soft Matter.

[3] Pasparakis G., Vamvakaki M. (2011). Multiresponsive polymers: Nano-sized assemblies, stimuli-sensitive gels and smart surfaces. Polym. Chem..

[4] Kopeček J., Yang J.Y. (2007). Hydrogels as smart biomaterials. Polym. Int..

[5] Osada Y., Gong J. (1993). Stimuli-responsive polymer gels and their application to chemomechanical systems. Prog. Polym. Sci..

[6] Roy D., Brooks W.L.A., Sumerlin B.S. (2013). New directions in thermoresponsive polymers. Chem. Soc. Rev..

[7] Liu R.X., Fraylich M., Saunders B.R. (2009). Thermoresponsive copolymers: From fundamental studies to applications. Colloid Polym. Sci..

[8] Beck J.B., Rowan S.J. (2003). Multistimuli, multiresponsive metallo-supramolecular polymers. J. Am. Chem. Soc..

[9] Noro A., Matsushita Y., Lodge T.P. (2008). Thermoreversible supramacromolecular ion gels via hydrogen bonding. Macromolecules.

[10] Jochum F.D., Theato P. (2013). Temperature- and light-responsive smart polymer materials. Chem. Soc. Rev..

[11] Burnworth M., Tang L.M., Kumpfer J.R., Duncan A.J., Beyer F.L., Fiore G.L., Rowan S.J., Weder C. (2011). Optically healable supramolecular polymers. Nature.

[12] Zhang Q., Qu D.-H., Wu J., Ma X., Wang Q., Tian H. (2013). A dual-modality photoswitchable supramolecular polymer. Langmuir.

[13] Dai S., Ravi P., Tam K.C. (2008). pH-Responsive polymers: Synthesis, properties and applications. Soft Matter.

[14] Charbonneau C.L., Chassenieux C., Colombani O., Nicolai T. (2011). Controlling the dynamics of self-assembled triblock copolymer networks via the pH. Macromolecules.

[15] Yao X., Chen L., Chen X., He C., Zhang J., Chen X. (2014). Metallo-supramolecular nanogels for intracellular pH-responsive drug release. Macromol. Rapid. Commun..

[16] Ten Brinke G., Ruokolainen J., Ikkala O. (2007). Supramolecular materials based on hydrogen-bonded polymers. Adv. Polym. Sci..

[17] Cordier P., Tournilhac F., Soulie-Ziakovic C., Leibler L. (2008). Self-healing and thermoreversible rubber from supramolecular assembly. Nature.

[18] Faul C.F., Antonietti M. (2003). Ionic self-assembly: Facile synthesis of supramolecular materials. Adv. Mater..

[19] Faul C.F.J. (2014). Ionic self-assembly for functional hierarchical nanostructured materials. Acc. Chem. Res..

[20] Hoeben F.J.M., Jonkheijm P., Meijer E.W., Schenning A.P.H.J. (2005). About supramolecular assemblies of π-conjugated systems. Chem. Rev..

[21] Brassinne J., Fustin C.-A., Gohy J.-F. (2013). Polymer gels constructed through metal-ligand coordination. J. Inorg. Organomet. Polym. Mater..

[22] Goshe A.J., Crowley J.D., Bosnich B. (2001). Supramolecular recognition: Use of cofacially disposed bis-terpyridyl square-planar complexes in self-assembly and molecular recognition. Helv. Chim. Acta.

[23] Goshe A.J., Steele I.M., Ceccarelli C., Rheingold A.L., Bosnich B. (2002). Supramolecular recognition: On the kinetic lability of thermodynamically stable host-guest association complexes. Proc. Natl. Acad. Sci. USA.

[24] Paulusse J.M.J., Huijbers J.P.J., Sijbesma R.P. (2006). Quantification of ultrasound-induced chain scission in Pd^II^–phosphine coordination polymers. Chem. Eur. J..

[25] Heskins M., Guillet J.E. (1968). Solution properties of poly(*N*-isopropylacrylamide). J. Macromol. Sci. Chem..

[26] Plamper F.A., Ruppel M., Schmalz A., Borisov O., Ballauff M., Muller A.H.E. (2007). Tuning the thermoresponsive properties of weak polyelectrolytes:  Aqueous solutions of star-shaped and linear poly(*N*,*N*-dimethylaminoethyl methacrylate). Macromolecules.

[27] Li F.-M., Chen S.-J., Du F.-S., Wu Z.-Q., Li Z.-C. (1999). Stimuli-responsive behavior of *N,N*-dimethylaminoethyl methacrylate polymers and their hydrogels. ACS Symp. Ser..

[28] Liu Q., Yu Z., Ni P. (2004). Micellization and applications of narrow-distribution poly[2-(dimethylamino)ethyl methacrylate]. Colloid Polym. Sci..

[29] Butun V., Armes S., Billingham N. (2001). Synthesis and aqueous solution properties of near-monodisperse tertiary amine methacrylate homopolymers and diblock copolymers. Polymer.

[30] He Y., Lodge T.P. (2008). Thermoreversible ion gels with tunable melting temperatures from triblock and pentablock copolymers. Macromolecules.

[31] Sugihara S., Kanaoka S., Aoshima S. (2004). Stimuli-responsive ABC triblock copolymers by sequential living cationic copolymerization: Multistage self-assemblies through micellization to open association. J. Polym. Sci. Part A Polym. Chem..

[32] Dyakonova M.A., Stavrouli N., Popescu M.T., Kyriakos K., Grillo I., Philipp M., Jaksch S., Tsitsilianis C., Papadakis C.M. (2014). Physical hydrogels via charge driven self-organization of a triblock polyampholyte—Rheological and structural investigations. Macromolecules.

[33] Piogé S., Fustin C.-A., Gohy J.-F. (2012). Temperature-responsive aqueous micelles from terpyridine end-capped poly(*N*-isopropylacrylamide)-*block*-polystyrene diblock copolymers. Macromol. Rapid. Commun..

[34] Brassinne J., Poggi E., Fustin C.-A., Gohy J.-F. (2015). Synthesis and self-assembly of terpyridine end-capped poly(*N*-isopropylacrylamide)-*block*-poly(2-(dimethylamino)ethyl methacrylate) diblock copolymers. Macromol. Rapid Commun..

[35] Mugemana C., Guillet P., Fustin C.-A., Gohy J.-F. (2011). Metallo-supramolecular block copolymer micelles: Recent achievements. Soft Matter.

[36] Brassinne J., Stevens A.M., van Ruymbeke E., Gohy J.-F., Fustin C.-A. (2013). Hydrogels with dual relaxation and two-step gel–sol transition from heterotelechelic polymers. Macromolecules.

[37] Jochum F.D., Brassinne J., Fustin C.-A., Gohy J.-F. (2013). Metallo-supramolecular hydrogels based on copolymers bearing terpyridine side-chain ligands. Soft Matter.

[38] Mugemana C., Joset A., Guillet P., Appavou M.-S., de Souza N., Fustin C.-A., Leyh B., Gohy J.-F. (2013). Structure of metallo-supramolecular micellar gels. Macromol. Chem. Phys..

[39] Guillet P., Mugemana C., Stadler F.J., Schubert U.S., Fustin C.-A., Bailly C., Gohy J.-F. (2009). Connecting micelles by metallo-supramolecular interactions: Towards stimuli responsive hierarchical materials. Soft Matter.

[40] Brassinne J., Gohy J.-F., Fustin C.-A. (2014). Controlling the cross-linking density of supramolecular hydrogels formed by heterotelechelic associating copolymers. Macromolecules.

[41] Brassinne J., Mugemana C., Guillet P., Bertrand O., Auhl D., Bailly C., Fustin C.-A., Gohy J.-F. (2012). Tuning micellar morphology and rheological behaviour of metallo-supramolecular micellar gels. Soft Matter.

[42] Brassinne J., Fustin C.-A., Gohy J.-F. (2013). Thermo-responsive metallo-supramolecular gels based on terpyridine end-functionalized amphiphilic diblock copolymers. Mater. Res. Soc. Symp. Proc..

[43] Brassinne J., Bourgeois J.-P., Fustin C.-A., Gohy J.-F. (2014). Thermo-responsive properties of metallo-supramolecular block copolymer micellar hydrogels. Soft Matter.

[44] Yan X., Wang F., Zheng B., Huang F. (2012). Stimuli-responsive supramolecular polymeric materials. Chem. Soc. Rev..

[45] Holyer R.H., Hubbard C.D., Kettle S.F.A., Wilkins R.G. (1966). The kinetics of replacement reactions of complexes of the transition metals with 2,2',2"-terpyridine. Inorg. Chem..

[46] Hogg R., Wilkins R. (1962). Exchange studies of certain chelate compounds of the transitional metals. Part VIII. 2,2',2"-terpyridine complexes. J. Chem. Soc..

[47] Schubert U., Hofmeier H., Newkome G.R. (2006). Modern Terpyridine Chemistry.

[48] Schubert U., Winter A., Newkome G.R. (2011). Terpyridine-Based Materials: For Catalytic, Optoelectronic and Life Science Applications.

[49] Aamer K.A., Tew G.N. (2007). Supramolecular polymers containing terpyridine-metal complexes in the side chain. Macromolecules.

[50] Ott C., Ulbricht C., Hoogenboom R., Schubert U.S. (2012). Metallo-supramolecular materials based on amine-grafting onto polypentafluorostyrene. Macromol. Rapid. Commun..

[51] Hackelbusch S., Rossow T., van Assenbergh P., Seiffert S. (2013). Chain dynamics in supramolecular polymer networks. Macromolecules.

[52] Calzia K.J., Tew G.N. (2002). Methacrylate polymers containing metal binding ligands for use in supramolecular materials: Random copolymers containing terpyridines. Macromolecules.

[53] El-ghayoury A., Hofmeier H., de Ruiter B., Schubert U.S. (2003). Combining covalent and noncovalent cross-linking: A novel terpolymer for two-step curing applications. Macromolecules.

[54] Hofmeier H., Schubert U.S. (2003). Supramolecular branching and crosslinking of terpyridine-modified copolymers: Complexation and decomplexation studies in diluted solution. Macromol. Chem. Phys..

[55] Brassinne J., Jochum F., Fustin C.-A., Gohy J.-F. (2015). Revealing the supramolecular nature of side-chain terpyridine-functionalized polymer networks. Int. J. Mol. Sci..

[56] Ott C., Lohmeijer B.G.G., Wouters D., Schubert U.S. (2006). Terpyridine-terminated homo and diblock copolymer LEGO units by nitroxide-mediated radical polymerization. Macromol. Chem. Phys..

[57] Lohmeijer B.G.G., Schubert U.S. (2005). The LEGO toolbox: Supramolecular building blocks by nitroxide-mediated controlled radical polymerization. J. Polym. Sci. Part A Polym. Chem..

[58] Keddie D.J. (2014). A guide to the synthesis of block copolymers using reversible-addition fragmentation chain transfer (RAFT) polymerization. Chem. Soc. Rev..

[59] Liu L., Wu C., Zhang J., Zhang M., Liu Y., Wang X., Fu G. (2008). Controlled polymerization of 2-(diethylamino)ethyl methacrylate and its block copolymer with *N*-isopropylacrylamide by RAFT polymerization. J. Polym. Sci. Part A Polym. Chem..

[60] Nuopponen M., Ojala J., Tenhu H. (2004). Aggregation behaviour of well defined amphiphilic diblock copolymers with poly(*N*-isopropylacrylamide) and hydrophobic blocks. Polymer.

[61] Rossow T., Habicht A., Seiffert S. (2014). Relaxation and dynamics in transient polymer model networks. Macromolecules.

[62] Rossow T., Seiffert S. (2014). Supramolecular polymer gels with potential model-network structure. Polym. Chem..

[63] Chiper M., Meier M.A.R., Kranenburg J.M., Schubert U.S. (2007). New insights into nickel(II), iron(II), and cobalt(II) bis-complex-based metallo-supramolecular polymers. Macromol. Chem. Phys..

[64] Vermonden T., van Steenbergen M.J., Besseling N.A.M., Marcelis A.T.M., Hennink W.E., Sudhölter E.J.R., Stuart M.A.C. (2004). Linear rheology of water-soluble reversible neodymium(III) coordination polymers. J. Am. Chem. Soc..

[65] Nicolai T., Colombani O., Chassenieux C. (2010). Dynamic polymeric micelles *versus* frozen nanoparticles formed by block copolymers. Soft Matter.

[66] Van Stam J., Creutz S., de Schryver F.C., Jérôme R. (2000). Tuning of the exchange dynamics of unimers between block copolymer micelles with temperature, cosolvents, and cosurfactants. Macromolecules.

[67] Wang Y., Balaji R., Quirk R.P., Mattice W.L. (1992). Detection of the rate of exchange of chains between micelles formed by diblock copolymers in aqueous solution. Polym. Bull..

[68] Jacquin M., Muller P., Talingting-Pabalan R., Cottet H., Berret J., Futterer T., Theodoly O. (2007). Chemical analysis and aqueous solution properties of charged amphiphilic block copolymers PBA-*b*-PAA synthesized by MADIX^®^. J. Colloid Interface Sci..

[69] Farina R.D., Hogg R., Wilkins R.G. (1968). Rate-pH profile for the dissociation of iron(II)-and cobalt(II)-2,2',2"-terpyridine complexes. Inorg. Chem..

[70] Mugemana C., Guillet P., Hoeppener S., Schubert U.S., Fustin C.-A., Gohy J.-F. (2010). Metallo-supramolecular diblock copolymers based on heteroleptic cobalt(III) and nickel(II) bis-terpyridine complexes. Chem. Commun..

[71] Mugemana C., Gohy J.-F., Fustin C.-A. (2012). Functionalized nanoporous thin films from metallo-supramolecular diblock copolymers. Langmuir.

[72] Fielding L.A., Lane J.A., Derry M.J., Mykhaylyk O.O., Armes S.P. (2014). Thermo-responsive diblock copolymer worm gels in non-polar solvents. J. Am. Chem. Soc..

[73] Dreiss C.A. (2007). Wormlike micelles: Where do we stand? Recent developments, linear rheology and scattering techniques. Soft Matter.

[74] Clausen T., Vinson P., Minter J., Davis H., Talmon Y., Miller W. (1992). Viscoelastic micellar solutions: Microscopy and rheology. J. Phys. Chem..

[75] Tam K., Jenkins R., Winnik M., Bassett D. (1998). A structural model of hydrophobically modified urethane-ethoxylate (HEUR) associative polymers in shear flows. Macromolecules.

[76] Howe A.J., Howe A.M., Routh A.F. (2011). The viscosity of dilute poly(*N*-isopropylacrylamide) dispersions. J. Colloid Interface Sci..

[77] Porter M.R. (1991). Handbook of Surfactants.

